# Resignation

**Published:** 1883-04

**Authors:** L. H. Hunt

**Affiliations:** New York


### RESIGNATION.

The medical editor regrets that he is obliged to discontinue his connection with The Independent Practitioner; but this is rendered necessary by the pressure of other work which has a prior claim. The fact that he is already in arrears with various duties on account of months of sickness in his family, and that it would be unfair to the readers of The Independent Practitioner as well as to the Columbia Publishing Co., for work to be done in a hurried style, which would be derogatory to this journal's standpoint—all these causes combined compel him (though very reluctantly) to give up his position.

The former genial editor, Dr. Wilkerson, has been very kind in aiding nearly all the work done since January, and it would be ungraceful not thus publicly to thank him.

Those who have contributed to the columns of The Independent Practitioner are tendered our hearty thanks. Those who have supported the paper hitherto will, we trust, support it yet more energetically in the future.

L. H. HUNT.

New York, April 25, 1883.

